# Temperature-attributable mortality projections under scenarios of climate change for Oslo, Norway

**DOI:** 10.1186/s12889-025-25980-3

**Published:** 2026-01-12

**Authors:** Liliana Vázquez Fernández, Alfonso Diz-Lois Palomares, Shilpa Rao, Ana María Vicedo-Cabrera

**Affiliations:** 1 Department of Biostatistics, Institute of Basic Medical Sciences, University of Oslo, Oslo, Norway; 2 Norwegian Institute of Public Health, Oslo, Norway; 3 Department of Mathematics, University of Oslo, Oslo, Norway; 4 Institute of Social and Preventive Medicine, University of Bern, Bern, Switzerland; 5 Oeschger Center for Climate Change Research, University of Bern, Bern, Switzerland

**Keywords:** Temperature, Environmental epidemiology, Mortality, Representative concentration pathways, Distributed lag non-linear model, Heat and cold

## Abstract

**Background & Aim:**

Climate change and evolving of population dynamics, including ageing and changes in population size, are reshaping temperature-attributable mortality patterns. However, there is limited evidence on the prospective trajectory of heat- and cold-attributable mortality in Oslo, particularly under combined scenarios of global warming and population development. This study aims to project heat- and cold-attributable mortality in Oslo and assess the distinct contributions of each of these drivers, utilising high-resolution data.

**Methods:**

We conducted a two-step approach with time series analysis with distributed lag non-linear models to estimate heat- and cold-attributable mortality relationship based on mean daily ambient temperature. Then, we performed a health impact assessment to compute the attributable mortality to heat and cold in the baseline period (2010–2019) and by the end of the century using regional population projections, mortality rates and projected daily temperature under two climate scenarios: RCP4.5 and RCP8.5.

**Results:**

For the RCP4.5/Medium Road scenario, the attributable mortality fractions for heat and cold are projected to increase over time, with values ranging from 9.05% (95%CI: 1.55–15.90) in 2010–2019 to 9.78% (95% CI: 2.96–15.86) in 2090–2099. Cold mortality consistently dominates the total, while heat mortality remains relatively low, starting at 1.80% (95%CI: 0.10–3.68) at baseline and increasing slightly to 3.12% (95%CI: 0.34–5.94) by the end of the century. In contrast, the RCP8.5/Strong Ageing scenario shows a more pronounced rise, with temperature-attributable mortality increasing from 9.07% (95%CI: 1.53–15.89) in 2010–2019 to 11.86% (95%CI: 4.29–18.53) in 2090–2099. In this scenario, heat mortality contributes significantly more, rising from 1.83% (95%CI: 0.12–3.85) in 2010–2019 to 5.99% (95%CI: 1.23–10.35) by 2090–2099, reflecting the greater climate and population impact under RCP8.5 and the Strong Ageing pathway.

**Conclusions:**

Our findings highlight the need for climate and population dynamics to be considered in public health policies. Tailored interventions are crucial to mitigate heat and cold-attributable mortality, particularly for vulnerable populations. Future research should integrate socio-economic factors and explore adaptation strategies to refine mortality projections and inform policy.

**Supplementary Information:**

The online version contains supplementary material available at 10.1186/s12889-025-25980-3.

## Introduction

Climate change-driven temperature changes are a key health risk in Europe both in the near and long term, with demonstrated increases in morbidity and mortality [1, 2]. Among the Nordic countries, Norway experiences wide variation in climatic conditions and faces challenging weather conditions [3]. Research suggests that Northern Europe is likely to experience an overall decline in temperature-attributable mortality in the short to medium term, as the reduction in cold-attributable deaths is expected to outweigh the rise in heat-attributable mortality [4, 5]. However, the health risks associated with temperature in Nordic regions remain insufficiently understood, particularly regarding the effects of an ageing and increasingly frail population.

In Norway, the proportion of the ageing population is projected to rise substantially. By the end of the century, under a strong ageing scenario, one in three Norwegians will be over 75, while children and adolescents will account for just one in ten [6]. These demographic shifts could exacerbate the health burden associated with non-optimal temperatures, as older adults are more vulnerable to both heat- and cold-attributable mortality.

Although no studies have specifically focused on the projected temperature-attributable health effects in Norway, a recent multi-city study that includes Oslo has examined these effects within a broader European context [7], alongside another assessing European regional differences [8]. However, research on the health impacts of rising temperatures in Norway and other Nordic countries remains limited. Historical studies have assessed temperature-attributable health effects in Denmark [9], Sweden [10–12] and Finland [13–16]. However, projections remain scarce, with a few exceptions, such as a recent study on six Finnish cities [17] and one on Sweden [18]. Some recent studies have explored future impacts in a broader European context, including Denmark [19], as well as projections for Copenhagen and Helsinki [5].

In Norway, previous research has focused on historical associations between temperature and health [2, 20, 21], but to date, no study has exclusively examined future projections exclusively in a Norwegian setting. This study presents, for the first time, quantitative projections of future attributable mortality due to temperature changes in Oslo under combined climate and demographic scenarios. The primary objective of this study is to assess changes in mortality due to temperature in Oslo under future scenarios of climate change and demographic change. To our knowledge, this is the first comprehensive study of this kind within Norway.

## Methods

We quantified the cold- and heat- related mortality burden in Oslo under various climate change and population development scenarios using a two-step approach, as described in Vicedo-Cabrera et al. (2019). First, we derived the temperature-mortality association for two age groups (using a 75-year cutoff) in Oslo [2]. This initial step involved a two-stage time-series analysis with first a quasi-Poisson regression model;

$$\:log\left\{E\right[{Y}_{t}\boldsymbol{}\left]\right\}=\alpha\:+cb({T}_{t}\boldsymbol{};\beta\:)+ns({time}_{t}\boldsymbol{},df=8\:per\:year)+{dow}_{t}$$

Where $\:{Y}_{t}\boldsymbol{}$ is the observed number of deaths in a day t, $\:E\left[{Y}_{t}\boldsymbol{}\right]$ its expected value, α the intercept, $\:cb({T}_{t}\boldsymbol{};\beta\:)$the distributed lag non-linear model (DLNM) cross-basis term modelling non-linear exposure-response and distributed lag structure for daily mean temperature $\:{T}_{t}\boldsymbol{}$ (parametrized by coefficients β), $\:ns({time}_{t}\boldsymbol{},df=8\:per\:year)$ a natural cubic spline of time to control for seasonality and long-term trends, and $\:{dow}_{t}$ an indicator for day of the day.

We chose eight degrees of freedom (df) per year for the time spline to remove seasonal and long-term trends while preserving the short-term associations of interest. Values between six and ten df per year are commonly used in temperature-mortality studies and eight df per year provides a balance between over- and under-smoothing. Three internal knots were placed at the 10th, 75th, and 90th percentiles of the temperature distribution to model the exposure-response dimension. This knot placement follows standard practice in the literature. Three equally spaced knots were used on the log scale for the lagged-response dimension, accounting for a 10-day lag period in line with previous studies.

We then reduced the bidimensional association to a one dimension overall cumulative temperature mortality association [23]. The model chosen specifications are based on previous studies [5]. Combinations of number of lags from 7 to 28 were tested and the best model was selected according to the epidemiological plausibility and empirical patterns observed in the exposure-response functions. Specifically, lag 10 represents the longest lag for which the estimated heat-attributable relative risks remain higher in the older age group (≥ 75 years) compared to the younger group (< 75 years), in line with the established vulnerability of older adults to heat exposure. Although shorter lags may underrepresent cold effects, the 10-day lag nonetheless captures a substantial proportion of the cold-attributable burden, providing a balanced and interpretable compromise for assessing temperature-attributable health risks across age groups. These differences are measured in terms of area under the curve (AUC) and presented on the Appendix (Table S1 and Figure S1).

From the exposure-response curves for each age group we derived the minimum mortality temperature (MMT), defined as the temperature at which the estimated mortality risk is lowest. We then used the MMT for each age group as the reference temperature for the estimation of the age-specific relative risks (RRs).

In the second step, we projected the cold- and heat- related mortality impacts for each group under two climate change scenarios using two representative concentration pathways (RCP) and two population development projections from Statistics Norway (SSB) [6]. Specifically, we utilised a combination of the two, coupling the RCP4.5 with the medium-migration, medium-fertility, and medium-mortality population projection from SSB, which we considered to represent a ‘Medium Road’ scenario, shared socioeconomic pathway (SSP2) [24]. SSP2 assumes medium projections for fertility, mortality, and migration. Similarly, we combined the RCP8.5 with a population projection characterised by strong population ageing, high fertility, and low mortality, corresponding to a ‘Strong Ageing’ scenario. This combination aligns with the assumptions of SSP5, marked by fossil-fuel driven economic development and demographic shifts [25].

We calculated the average annual attributable mortality (AN) and fractions (AF) per GCM and RCP/SSP, and age group. The fraction is defined as the AN divided by the total mortality in the period, expressed as a percentage. Cold-attributable mortality was considered as deaths occurring on days below the MMT, while heat-attributable mortality was defined as deaths occurring on days with temperatures above the MMT. The 95% empirical confidence intervals (CIs) were derived using Monte Carlo simulations. Specifically, we generated 1,000 simulations of the coefficients that define the exposure-response functions and derived a set of simulations representing the impacts for each GCM, age group, climate and population scenario. This approach ensure that the uncertainty estimates incorporate both variability across model and the imprecision in the association estimates [22].

Finally, we evaluated the contributions of progressive warming and population changes to the projected temperature-mortality impacts. We first estimated the temperature-attributable mortality burden under a ‘climate-only’ scenario by applying temperature projections for each climate scenario while keeping the population fixed at baseline levels. The contribution of climate warming was quantified as the difference between the impacts in the ‘climate-only’ scenario and the remaining burden, which was attributed to changes in population structure. Additionally, we compared each RCP with the two population development pathways to assess the relative contributions of the different socioeconomic scenarios.

To compare mortality burdens across decades, we calculated differences relative to the baseline decade of 2010–2019. For these comparisons, point estimates were obtained by subtracting the decadal impacts, and the corresponding uncertainty was quantified using empirical confidence intervals. These were derived directly from the Monte Carlo simulations, capturing the variability in the simulation results. Specifically, the distribution of differences was calculated by subtracting the simulated impacts of the baseline decade from those of the comparison decade across all simulations. The 95% empirical CIs were then determined by identifying the 2.5th and 97.5th percentiles of the resulting distribution of differences. This empirical approach reflects the uncertainty inherent in the simulations without relying on parametric assumptions [22].

### Temperature data

We obtained gridded data on daily maximum temperature for the period 1996–2100. For the historical period 1996–2014, we used data from the *seNorge* temperature dataset [26]. For the future period 2015 to 2100, we employed projections from EUROCORDEX climate simulations (Coupled Model Intercomparison Project—Phase 6 (CMIP6)) on a set of general circulation model (GCM) for RCP4.5 and RCP8.5 (i.e., 6 simulations, respectively) [27]. All data were extracted at the centroid of Oslo municipality, defined as latitude 59.98 and longitude 10.74. We constructed daily maximum temperature time series for each of the six GCMs for RCP4.5 and RCP8.5 respectively. The list of the GCM IDs is provided in Supplementary Table S2a. Furthermore, we bias-corrected the simulated temperature series using the observed temperature series following the method specified by Hempel et al. [28]. This correction adjusted the climate model data to align with the historical data by applying a constant offset while preserving long-term temperature differences. We evaluated the bias correction over the overlapping historical period (2000–2018). Across EURO‑CORDEX realisations the median mean model bias was + 1.63 °C (RCP4.5) and + 1.52 °C (RCP8.5) before correction; after applying the Hempel et al. correction the median residual bias was effectively zero (≈ − 0.001 °C for both scenarios). Full per-model diagnostics are provided in the Supplement (Supplementary Table S2a) and summary diagnostics (model-wise median and ensemble-median) are given in Supplementary Table S2b.

## Mortality data

The health data comprised daily counts for all non-accidental mortality in Norway (International Classification of Diseases, ICD-10: A00-R99) between 1 January 1996 and 31 December 2018, provided by the Norwegian Death Registry (Norwegian Cause of Death Registry), disaggregated by age. The registry provides national coverage and is the official source of cause‑of‑death statistics.

We accounted for changes in the demographic structure of the population by using the regional mortality and population size projections from Statistics Norway [29] for the period 2019 to 2050. For the years 2051 to 2100, we downscaled the national projections [30] based on the average percentages per age group observed between years 2016 and 2022. We then derived daily non-accidental deaths by age group, accounting for seasonality by using the share of the deaths by day of the year per age group from the observed mortality. For each age group we calculated the mean daily share of annual deaths for each calendar day by averaging the observed day-of-year death proportions across the historical period. Projected annual deaths by age group were then allocated to individual days using these mean daily shares, thereby preserving the observed seasonal pattern in the baseline while avoiding year-to-year noise. Projected annual deaths (2019–2100) were taken from Statistics Norway as reported (total deaths); the historical exposure–response was estimated on non-accidental deaths (ICD-10 A00–R99), and we assume the accidental fraction remains small and approximately constant over time. Subsequently, we combined the temperature series of each GCM and the historical mortality series by age group with the RCP4.5/Medium Road and RCP8.5/Strong Ageing to estimate the cold- and heat- related mortality impacts by age group, GCM, and decade. For clarity, the RCP4.5/Medium Road scenario couples a moderate radiative‑forcing pathway (RCP4.5) with SSB’s medium‑migration, medium‑fertility and medium‑mortality population projection, while the RCP8.5/Strong Ageing scenario couples a high‑forcing pathway (RCP8.5) with a population projection characterised by stronger ageing (higher proportion of older adults), higher fertility and lower mortality.

The baseline period is 2010–2019 and we report mortality impacts by decade with respect to the baseline period. We selected 2010–2019 as the baseline because it is the most recent decade immediately prior to the projection period and therefore best represents contemporary population structure, healthcare provision and reporting practices used in our century-long projections. Using a recent ten-year period balances recency with sampling of interannual variability and avoids confusing long-term historical declines in mortality with projected changes.

## Results

**Fig. 1:**
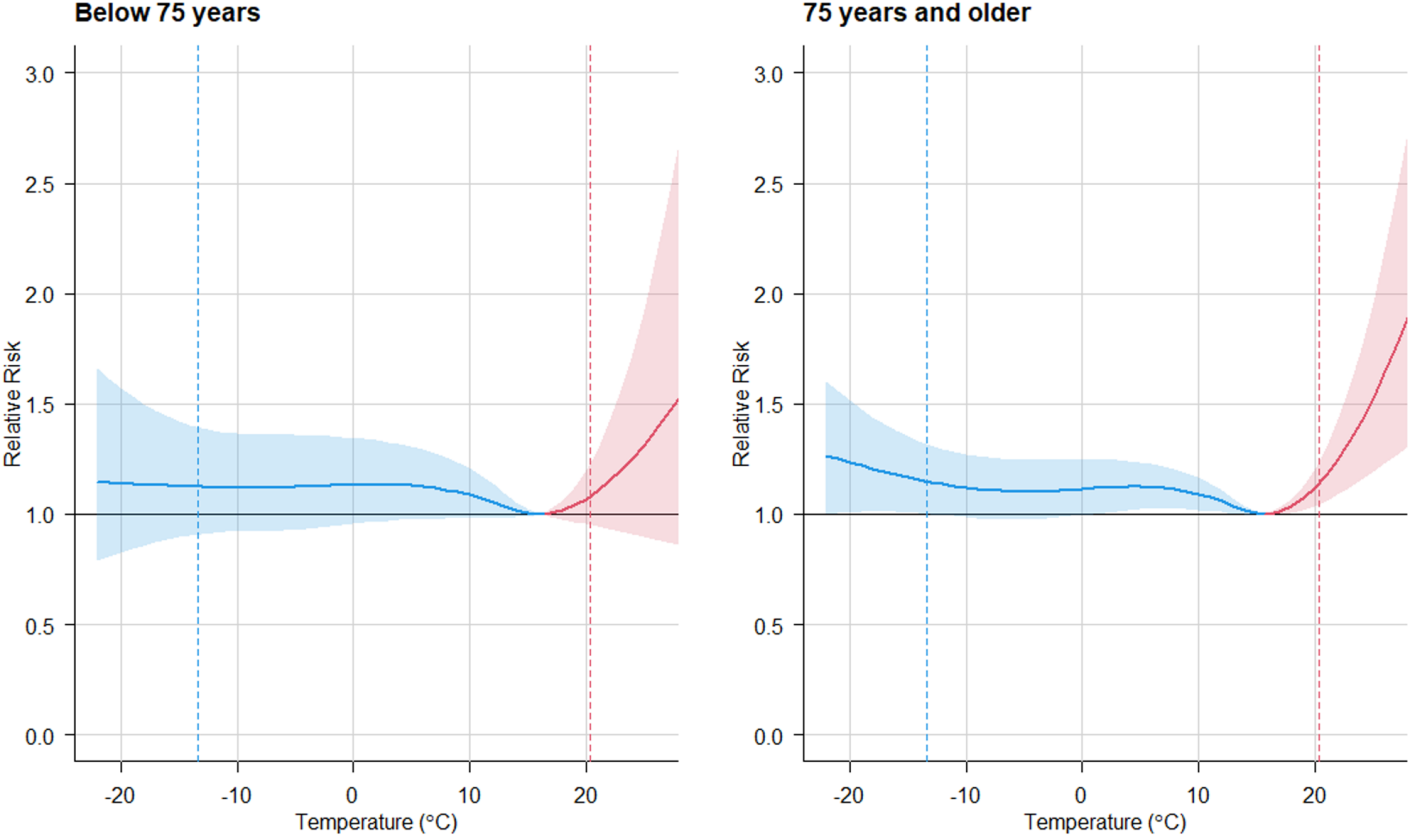
Overall cumulative exposure-response functions between temperature and non-accidental mortality by age group. Shaded areas on the curves are 95% CIs. Dashed lines correspond to the 1st and 99th percentiles of the temperature distribution

Figure 1 illustrates the overall temperature-mortality association for the period 1996–2014, stratified by age group. Relative risks (RR) due to cold are shown in blue, and those due to heat are shown in red. The older population, aged 75 years and above, exhibits greater vulnerability to high temperatures, with a RR of 1.14 (95%CI: 1.05–1.24) at the 99th percentile of the temperature distribution (20.4˚C) vs. MMT, compared to the group of under 75 years (RR 1.08 (95%CI: 0.95–1.23)). The cold-attributable risk is also greater in the older age group, with extreme cold defined at the 1 st percentile of the temperature distribution (−13.5˚C), with a RR of 1.15 (95%CI: 1.00–1.32.00.32), compared to the younger group (1.12 (95%CI: 0.91–1.39)).

**Fig. 2:**
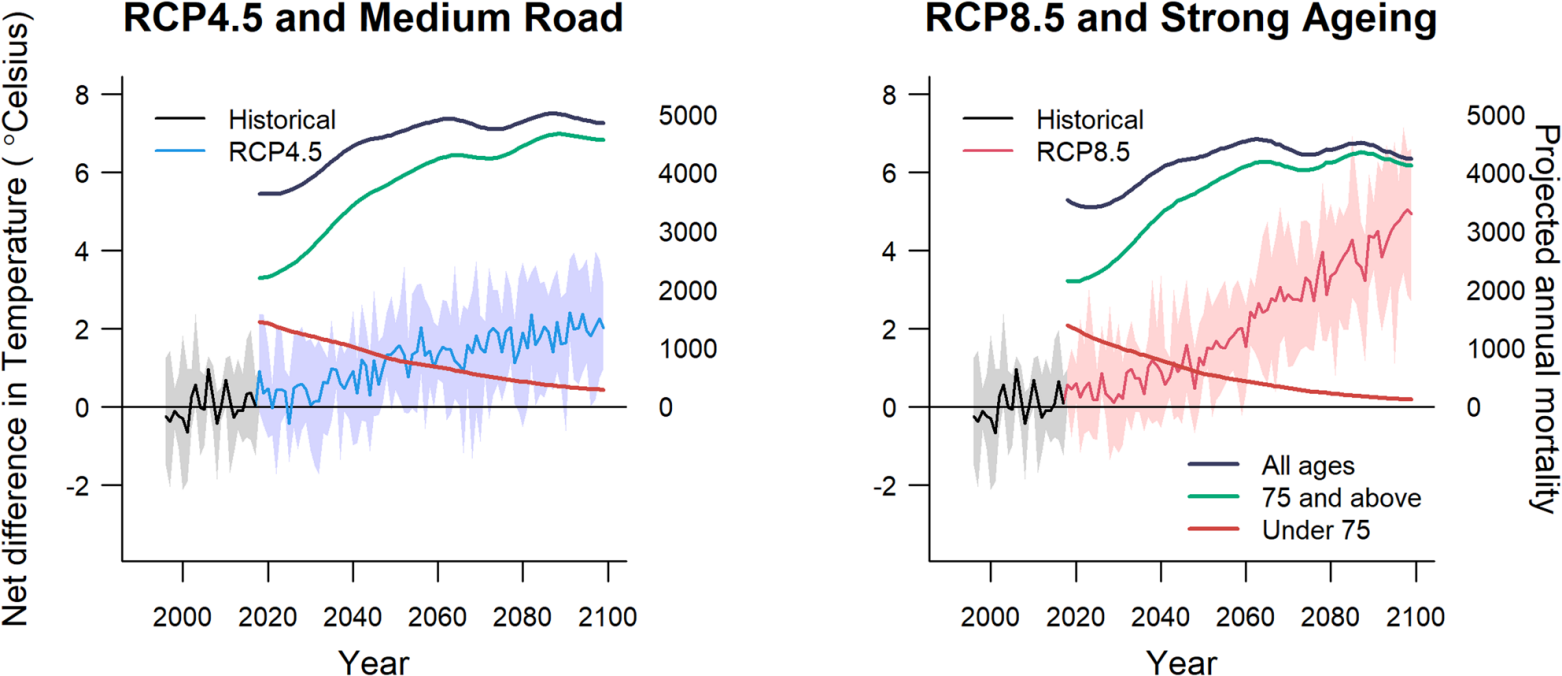
Projected annual maximum temperature an age-specific mortality trends by Representative Concentration Pathways (RCP) and Shared Socio-Economic Pathways Scenarios in Oslo

Figure 2 presents historical and projected temperatures and mortality under the different scenario combinations (RCP4.5/Medium Road and RCP8.5/Strong Ageing) by age group. The data for all ages are shown in dark blue, under 75 years in red, and 75 years and over in green. For both scenarios, overall mortality is projected to increase gradually over time. Mortality growth is slightly more pronounced in the Medium Road (SSP2) scenario compared to Strong Ageing (SSP5), particularly after 2025. Mortality in the under-75 age group is projected to decrease in both scenarios, with Strong Ageing (SSP5) showing a steeper reduction. For the older age group, the increase in mortality is larger in the Strong Ageing (SSP5) scenario, reflecting the pronounced ageing trend of the population under this pathway. As a result, the gap between mortality in the younger and older age groups widens more rapidly in Strong Ageing (SSP5) scenario, with more substantial increases in mortality among those aged 75 and over. This may reflect the heightened vulnerability of the elderly population to the assumed higher levels of stress and challenges in Strong Ageing (SSP5) scenario, including health and environmental factors.

With regards to the RCP, in the early years (2010–2019), the average values for the GCM under both RCP4.5 and RCP8.5 are relatively similar, with a slight increase observed under RCP8.5. Over time, the RCP4.5 shows a consistent rise from an annual temperature of 5.6˚C in 2019 to 7.5˚C in 2099, while RCP8.5 shows a more pronounced increase, beginning at 5.7˚C in 2019 and reaching 10.2˚C by 2099. The differences between the two RCP increase over the decades, starting with a difference of 0.1–0.2 °C in the early years, widening to 0.3–0.5 °C by mid-century, and becoming more significant in the late century, exceeding 1 °C and, in some years, approaching 2 °C by 2099.

The projected decadal attributable mortality related to heat and cold for both RCP4.5 Climate Only and RCP4.5/Medium Road scenarios (Table 1) remain relatively stable throughout the century, starting at 9.05%(95% CI: 1.55–15.90) and increasing slightly to 9.56% (95% CI: 1.59–16.43) and 9.78 (95% CI: 2.96–15.86), respectively. Cold-attributable temperature-attributable mortality dominate the burden, consistently accounting for more temperature-attributable deaths than heat-related mortality. However, heat-attributable temperature-attributable mortality show a gradual rise, increasing from 0.91% (95% CI: −0.13–2.48) in 2010–2019 to 2.80 (0.08–5.89) for Climate Only and to 3.12% (0.34–5.94) for RCP4.5/Medium Road.

**Table 1:** Projected decadal attributable mortality fractions related to heat and cold under RCP4.5 for different population development scenarios (Climate only and RCP4.5/Medium Road)

	RCP4.5 Climate Only			RCP4.5/Medium Road		
Decades	Total	Cold	Heat	Total	Cold	Heat
2010-19	9.05 (1.55–15.90)	7.26 (0.19–13.87)	1.80 (0.10–3.68)	9.05 (1.55–15.9)	7.26 (0.2–13.87.2.87)	1.80 (0.10–3.68)
2020-29	8.99 (1.14–16.06)	7.29 (−0.02-14.09)	1.70 (0.04–3.95)	9.00 (1.30–15.95.30.95)	7.28 (0.12–13.97)	1.72 (0.07–3.96)
2030-39	8.99 (1.15–16.02)	7.24 (−0.04-14.03)	1.75 (0.04–4.37)	9.03 (1.58–15.74)	7.20 (0.31–13.64)	1.83 (0.11–4.41)
2040-49	9.09 (1.39–15.96)	7.06 (0.02–13.68)	2.03 (0.09–4.50)	9.17 (2.08–15.54)	7.00 (0.54–13.12)	2.17 (0.28–4.56)
2050-59	9.26 (1.45–16.22)	6.92 (0.01–13.38)	2.34 (0.08–6.13)	9.38 (2.35–15.74)	6.85 (0.65–12.72)	2.54 (0.31–6.23)
2060-69	9.27 (1.51–16.09)	6.93 (0.02–13.38)	2.34 (0.13–5.01)	9.41 (2.59–15.58)	6.85 (0.75–12.66)	2.56 (0.44–5.08)
2070-79	9.27 (1.51–16.20)	6.79 (0.05–13.07)	2.48 (0.08–5.69)	9.44 (2.62–15.60)	6.70 (0.78–12.30)	2.74 (0.33–5.75)
2080-89	9.52 (1.59–16.64)	6.77 (−0.01-13.14)	2.75 (0.13–6.46)	9.72 (2.81–16.04)	6.67 (0.84–12.33)	3.05 (0.48–6.52)
2090-99	9.56 (1.59–16.43)	6.76 (0.07–13.10)	2.80 (0.08–5.89)	9.78 (2.96–15.86)	6.66 (0.93–12.19)	3.12 (0.34–5.94)

Under RCP8.5 (Table 2), a more pronounced increase in total and heat-attributable mortality is observed, particularly in the later decades of the century. By 2090–2099, the total temperature-attributable deaths rise to 11.32% (95% CI: 2.59–18.96) for Climate Only and 11.86% (95%CI: 4.29–18.53) for RCP8.5/Strong Ageing, compared to 9.07% (95%CI: 1.53–15.87) in 2010–2019. Heat-attributable mortality shows a sharp increase during this period, reaching 5.37% (95%CI: 0.42–10.35) in Climate Only and 5.99% (95%CI: 1.23–10.35) in RCP8.5/Strong Ageing, reflecting the combined effects of both higher emissions and socioeconomic growth. In contrast, cold-attributable temperature-attributable deaths show a consistent decline over the century, from 7.24% (95%CI: 0.16–13.86) in 2010–2019 to 5.94% (95%CI: 0.09–11.45) by 2090–2099 in Climate Only, and to 5.88% (95%CI: 1.00–10.70.00.70) in RCP8.5/Strong Ageing. These findings highlight the growing contribution of heat-attributable mortality under higher emissions scenarios and the steep decline of cold-attributable impacts to partially offset these trends.

**Table 2:** Projected decadal attributable mortality fractions related to heat and cold under RCP8.5 for different population development scenarios (Climate only and RCP8.5/Strong Ageing)

	RCP8.5 Climate Only			RCP8.5/Strong Ageing		
Decades	Total	Cold	Heat	Total	Cold	Heat
2010-19	9.06 (1.52–15.87)	7.24 (0.16–13.86)	1.83 (0.12–3.85)	9.07 (1.53–15.87)	7.24 (0.16–13.85)	1.83 (0.12–3.85)
2020-29	9.22 (1.35–16.34)	7.27 (−0.01-13.97)	1.95 (0.05–4.48)	9.25 (1.56–16.22)	7.25 (0.14–13.82)	1.99 (0.10–4.49)
2030-39	9.26 (1.31–16.43)	7.17 (0.01–13.91)	2.08 (0.03–5.19)	9.33 (1.89–16.05)	7.12 (0.43–13.41)	2.21 (0.12–5.27)
2040-49	9.28 (1.38–16.35)	7.06 (−0.02-13.72)	2.23 (0.09–5.65)	9.39 (2.25–15.86)	6.99 (0.63–13.03)	2.41 (0.30–5.74)
2050-59	9.63 (1.56–16.94)	6.89 (0.03–13.25)	2.74 (0.12–6.42)	9.81 (2.70–16.33.70.33)	6.79 (0.76–12.51)	3.02 (0.44–6.50)
2060-69	10.30 (2.06–17.95)	6.64 (0.09–12.73)	3.66 (0.23–8.64)	10.59 (3.32–17.49)	6.55 (0.87–11.96)	4.04 (0.69–8.83)
2070-79	10.01 (1.94–17.11)	6.37 (0.05–12.28)	3.63 (0.22–7.46)	10.32 (3.38–16.63)	6.29 (0.92–11.48)	4.04 (0.75–7.57)
2080-89	10.76 (2.16–18.43)	6.18 (0.11–11.88)	4.58 (0.27–9.51)	11.20 (3.81–17.99)	6.10 (1.00–11.06.00.06)	5.10 (0.75–9.61)
2090-99	11.32 (2.59–18.96)	5.94 (0.09–11.45)	5.37 (0.42–10.35)	11.86 (4.29–18.53)	5.88 (1.00–10.70.00.70)	5.99 (1.23–10.35)

**Fig. 3:**
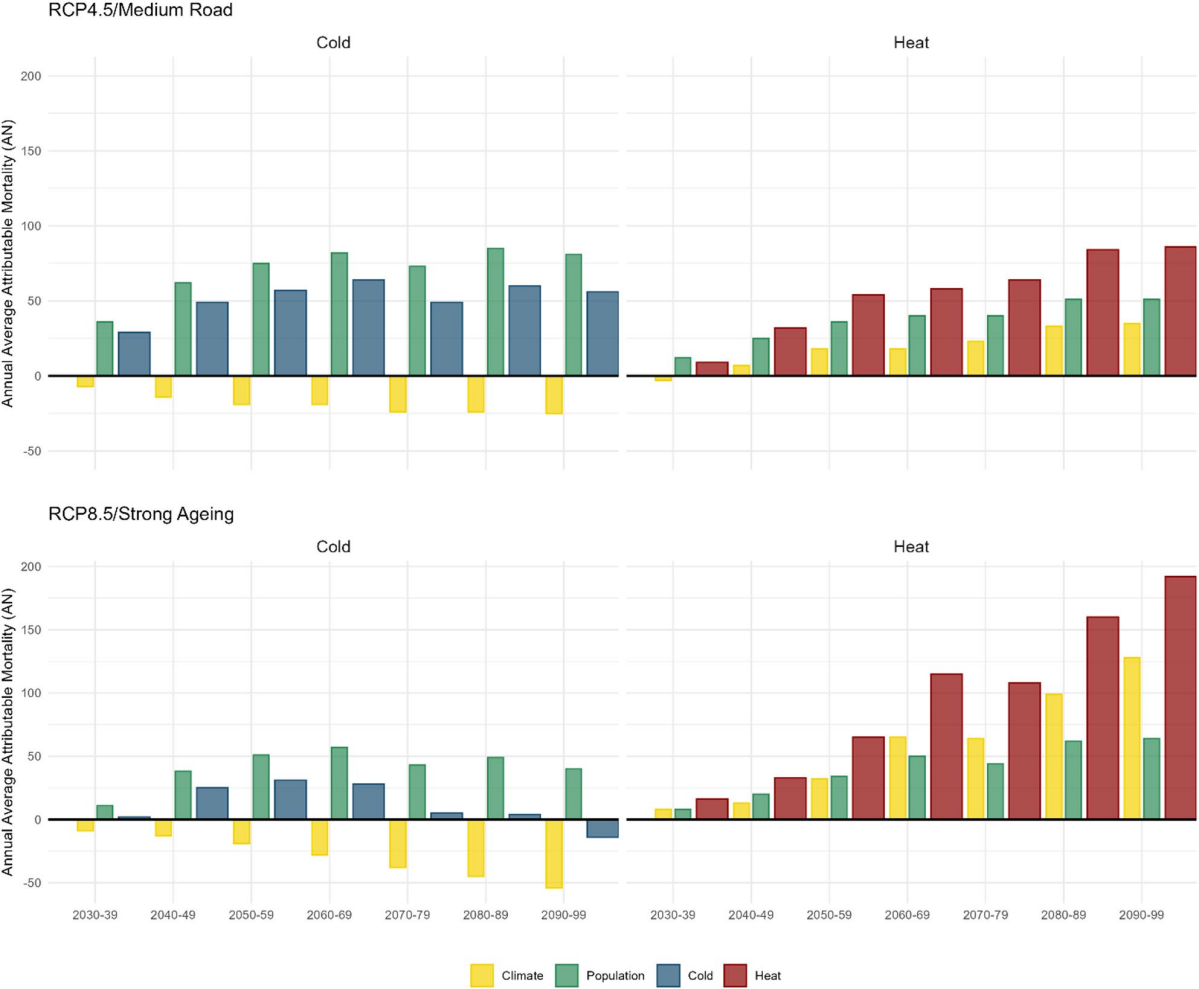
Projected annual average differences in heat- and cold-attributable mortality in Oslo per decade by RCP/SSP scenario and warming target, relative to the 2010–2019 baseline. The contributions from population changes are shown in green, climate impact in yellow, with overall heat-attributable mortality in red and cold-attributable mortality in blue

Figure 3 illustrates the average annual decadal contributions of climate and population changes to heat- and cold-attributable mortality under RCP4.5/Medium Road and RCP8.5/Strong Ageing scenarios. The trends under RCP4.5 highlight more moderate changes compared to the observed in RCP8.5. Under the RCP4.5 scenario, climate-driven contributions to cold-attributable mortality steadily decline over time, relative to 2010–2019, to −25 (95%CI: −64-6) by 2090–2099. Notably, these contributions remain negative throughout the century, reflecting a gradual decline in cold-attributable mortality. Conversely, climate-driven heat-attributable mortality declines until 2040-49 when it starts increasing from 7 (95%CI: −28-37) to 35 (95%CI: −4-144) by 2090–2099, though the increase is less pronounced compared to RCP8.5.

Population-driven changes also play a significant role under RCP4.5. For cold-attributable mortality, population changes lead to an increase, rising from 36 (95%CI: 33–40) in 2030–2039 to 81 (95%CI: 73–90) by 2090–2099. Similarly, population effects on heat-attributable mortality grow over time, 13 (95%CI: 3–22) in 2030–2039 to 51 (95%CI: 14–72) by 2090–2099, underscoring the demographic shifts that amplify heat-attributable risks. When combining climate and population effects, cold-attributable mortality under RCP4.5 rises from 29 (95%CI: 2–61) in 2030–2039, peaks at 61 (95%CI: 25–114) in 2060–2069, and declines slightly to 56 (95%CI: 27–102) by 2090–2099. In contrast, heat-attributable mortality shows a steady upward trend, growing from 9 (95%CI: −27-60) in 2030–2039 to 86 (95%CI: 11–191) by 2090–2099. These patterns suggest that while heat-attributable risks increase steadily, cold-attributable mortality declines more gradually under RCP4.5 than under RCP8.5.

Under the RCP8.5 scenario, the annual average contributions of climate and population changes to heat- and cold-attributable mortality, per decade, show similar trends to RCP4.5. The climate-driven contribution to cold-attributable mortality declines progressively throughout the century, similarly to RCP4.5 until 2050–2059 and from there, the reduction is progressively enlarging. The lowest value is reached in 2090–2099 (−54 (95%CI: −124-10)). In contrast, climate-driven heat-attributable mortality rises continuously from 8 (95%CI: −13-53) in 2030-39, and more sharply in 2060–2069 (65 (95%CI: 1–177)) to 127 (95%CI: 10–237) by 2090–2099, reflecting the impact of increasing temperatures.

Population-driven changes show a more nuanced pattern. Cold-attributable mortality attributable to population changes fluctuates, increasing until 2060–2069 (55 (95%CI: 51–61)) and stabilising hereafter. For heat-attributable mortality, population-driven contributions steadily increase from 8 (95%CI: 2–13) in 2030–2039 to 64 (95%CI: 26–83) in 2090–2099, indicating that demographic changes drive heat-attributable mortality. Considering overall differences in heat- and cold-attributable mortality relative to 2010–2019, cold-attributable temperature-attributable deaths decrease significantly over time, from 2 (95%CI: −24-19) in 2030–2039 to −13 (95%CI: −78-44) by 2090–2099. Meanwhile, heat-attributable mortality rises sharply from 16 (95%CI: −8-64) in 2030–2039 to 192 (95%CI: 46–308) by the 2090–2099, driven by both rising temperatures and demographic changes. These trends emphasise the growing burden of heat-attributable impacts, driven predominantly by climate change, and the diminishing contribution of cold-attributable mortality under RCP8.5.

Tables S3 and S4 provide the attributable fractions of attributable mortality disaggregated by the contribution of each driver for RCP4.5 and RCP8.5. Tables S5 and S6 show the net differences in projected annual average attributable mortality due to heat and cold under RCP4.5 and RCP8.5 per decade, relative to 2010–2019, which were used to generate Fig. 3. Tables S7 and S8 provide the net differences in attributable fraction (total, cold and heat) for both RCPs.

When combining RCP4.5 with the Strong Ageing scenario, we estimate that by the end of the century, the difference between RCP4.5/Medium Road and RCP4.5/Strong Ageing in heat-attributable mortality is an 18.9% decrease compared to RCP4.5/Medium Road, while the reduction in cold-attributable mortality is larger, at 65.5%. Under RCP8.5, the decrease in heat-attributable mortality is smaller (14%) while the reduction in cold-attributable mortality is substantial, at 175%. These results comparing Medium Road and Strong Ageing scenarios for RCP4.5 are included in Table S9 and for RCP8.5 in Table S10.

## Discussion

In this study, we provided an extensive comparative assessment of the non-optimal temperature mortality impacts per decade of the 21 st century in Oslo, Norway. We found a substantial increase in temperature-attributable mortality under all scenarios, with the overall burden ultimately dependant on the emission and population development pathway. Particularly, population ageing amplifies future non-optimal temperatures burdens. Notably, the patterns observed for heat- and cold-attributable mortality diverged, with the relative contribution of each varying markedly across scenarios and decades, underscoring the importance of disaggregating their respective impacts.

We observed that the scenario RCP4.5/Medium Road is projected to result in larger overall mortality, both cold- and heat- related, than RCP8.5/Strong Ageing. The total number of temperature-attributable deaths is higher in RCP4.5/Medium Road until the decade of 2060–2069, when the RCP8.5/Strong Ageing surpasses it. For cold-attributable mortality, RCP4.5/Medium Road remains higher than RCP8.5/Strong Ageing throughout the century. Conversely, heat-attributable mortality under RCP8.5/Strong Ageing, relative to 2010–2019, increases progressively over the decades, eventually doubling the number of temperature-attributable deaths by 2090–2099, (1915 (458–3081) vs. 858 (107–1907)).

Our results do not suggest a net reduction in temperature-attributable deaths for Oslo, as has been found in previous assessments for Northern Europe, although these assessments do not include Norway [5, 19]. Gasparrini et al. [5] reported a net excess mortality reduction for Stockholm, Sweden, of −0.7% (−2.1–0.3) for 2090-99 under RCP4.5 and − 0.4% (−3.1–2.3) under RCP8.5. A recent study encompassing 854 cities, including Oslo and three other Norwegian cities, also projected a net reduction by 2095, with an estimated − 3.9 deaths attributable to temperature per 100,000 person years under RCP3.7 [7]. In contrast, our analysis suggests a net increase in temperature-attributable mortality of Oslo, with an estimated net rise in the AF of 2.89% (1.02–4.92) under RCP4.5, and 4.11% (0.39–7.07) under RCP8.5, relative to the 2010–2019.

The general net increase in temperature-attributable mortality across scenarios can be explained by the shape of the exposure-response function. We find that the mortality risk increases more steeply at higher temperatures than it decreases at lower temperatures for both age groups, meaning that even a moderate warming of the temperature distribution would result in a net rise in deaths. Additionally, we obtained a MMT of 16˚C for Oslo that corresponds to a high minimum mortality percentile, around 92nd percentile of the temperature distribution. This suggests that while Oslo’s population may have some resilience to moderate warmth, it is vulnerable to rising heat temperatures [7]. Although in terms of cold-attributable mortality, our results show that cold impacts will increase under RCP4.5/Medium Road [31], while they remain relatively unchanged under RCP8.5/Strong Ageing [32]. As a result, the overall net increase in temperature-attributable mortality is notable. This also highlights the imperative need of adaptation to a warming world. Norway faces several challenges in effectively managing and mitigation climate changes risks, including a fragmented responsibility structure, inadequate cross-sector coordination, a lack of systematic risks assessments, and insufficient systems for monitoring and evaluation [3]. In this context, adaptation strategies must integrate both physiological and socioeconomic measures to reduce temperature-attributable mortality. While physiological acclimatisation may occur over time, the role of socioeconomic adaptation, through improvements in healthcare infrastructure, early warning systems, and sustainable heating and cooling strategies [33], will be critical in strengthening Norway’s resilience to both heat and cold-attributable risks [34]. Many adaptation measures deliver simultaneous health and climate co‑benefits: improving housing thermal performance (insulation, shading, ventilation and efficient heating/cooling) can reduce cold‑ and heat‑related mortality, lower energy consumption and reduce fuel poverty; pairing insulation with ventilation and shading is important to avoid unintended summertime overheating.

Our municipal‑level estimates are directly relevant to local public‑health and adaptation planning in Norway’s decentralised governance context. By providing city‑scale projections of heat‑ and cold‑related mortality by age group and decade, the results can inform municipal-level operations such as setting local heat action plans, prioritising neighbourhoods and age groups for outreach and cooling interventions, aligning seasonal capacity planning in emergency and primary care, and integrating climate‑health risks into municipal risk assessments, land‑use decisions and building‑code updates. Because municipalities in Norway are responsible for primary healthcare, eldercare and local emergency response, these localised burden estimates enable evidence‑based targeting of resources and the monitoring of adaptation effectiveness over time.

A strength of this study is the use of the new CMIP6 simulations, instead of the CMIP5, which a recent investigation has found to underestimate the magnitude of warming in Europe, although for Northern Europe this difference is smaller than in the South [35]. We included changes in mortality and population structure to project temperature-attributable mortality impacts. Conversely, we used time-invariant exposure-response functions, meaning the impacts do not account for changes in vulnerability to heat and cold, assuming that there is no future adaptation in the decades to come. A key limitation of this study is the exclusion of adaptation mechanisms, which have been shown to significantly influence temperature-attributable mortality projections [36], particularly given the large scale of the projections; which may overestimate the temperature-attributable mortality projections [37]. Strategies such as shifts in minimum mortality temperature, changes in relative risk, and improvements in socioeconomic adaptive capacity could alter future burden estimates. Future work should integrate these factors to enhance the accuracy and policy relevance of projections.

Our study only relied on two age categories (under 75 years and 75 and above) due to a lack of statistical power to have more disaggregated age groups. Nonetheless, we consider that our study captures the changes in vulnerability and structure of the population, as the predominant effects of temperature occur in the older populations. We did not analyse neighbourhood‑level variation in this study. Finer spatial analyses at sub‑municipal scale would be valuable future work to identify local hotspots and target interventions more precisely.

## Conclusion

Our findings indicate that mortality associated with non-optimal temperatures will increase across all climate change scenarios examined. The combined effects of population dynamics and rising temperatures will lead to a greater heat-attributable burden in Oslo, which will not be counterbalanced by a reduction in cold-attributable mortality. These results highlight the urgent need to establish measures and resources for monitoring and mitigating the impacts of climate change and protect public health.

## Supplementary Information

Below is the link to the electronic supplementary material.

Supplementary Material 1.

## Data Availability

The individual mortality data used in this study were obtained from the Norwegian Cause of Death Registry (Dødsårsaksregisteret, DÅR), maintained by the Norwegian Institute of Public Health (NIPH). These data are confidential and cannot be shared by the authors. Interested researchers may request access directly from DÅR via NIPH. Mortality projections were obtained from Statistics Norway (SSB) and are publicly available. Daily historical temperature data were sourced from the seNorge_2018 dataset, while climate projection data were retrieved from the CMIP6 archive via the Copernicus Climate Data Store.

## Abbreviations

AF: Attributable Fraction
AN: Attributable Number
AUC: Area Under the Curve
CI: Confidence Interval
CMIP6: Coupled Model Intercomparison Project Phase 6
GCM: General Circulation Model
MMT: Minimum Mortality Temperature
RCP: Representative Concentration Pathway
RR: Relative Risk
SSB: Statistics Norway (Statistisk sentralbyrå)
SSP: Shared Socioeconomic Pathway
