# Supplementary material for: Temperature-attributable mortality projections under scenarios of climate change for Oslo, Norway
Source: BMC Public Health. 2026 Jan 12;26:511. doi: 10.1186/s12889-025-25980-3 (PMC12888328; doi:10.1186/s12889-025-25980-3)
Supplement: Supplementary file 1 — Supplementary Material 1. [file 12889_2025_25980_MOESM1_ESM.docx]

**Temperature-attributable mortality projections under scenarios of climate change for Oslo, Norway**

**Figure S1.** Sensitivity analysis of the temperature–mortality exposure–response relationships by age group and lag structure in Oslo.

Estimated relative risks associated with temperature across lag structures (lags 7, 10, 14, and 21 days) for individuals aged below 75 years (left) and those aged 75 years and older (right). The upper panel presents the exposure–response curves without confidence intervals, while the lower panel includes 95% empirical confidence intervals. Results illustrate lag-dependent variations.


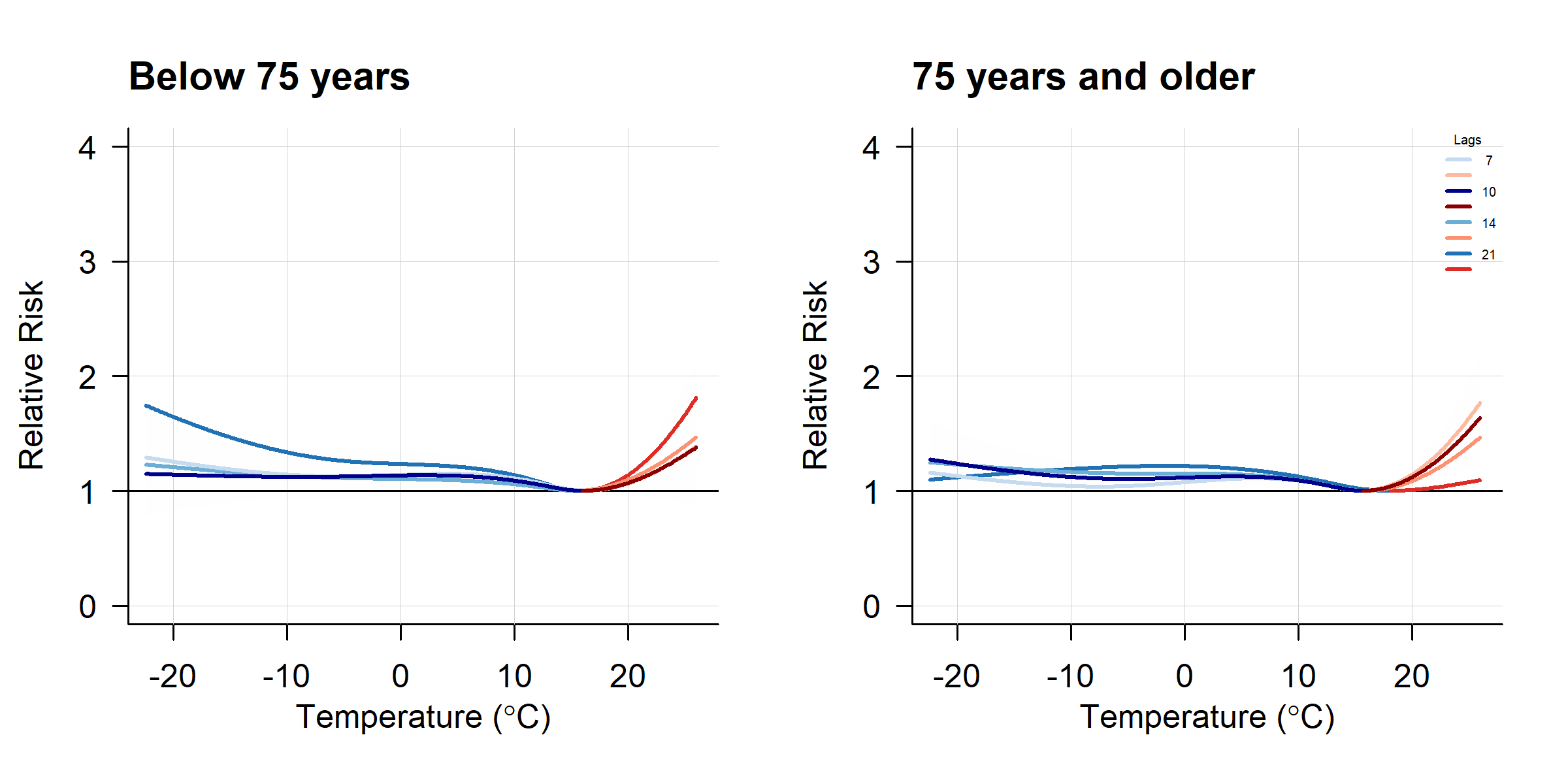


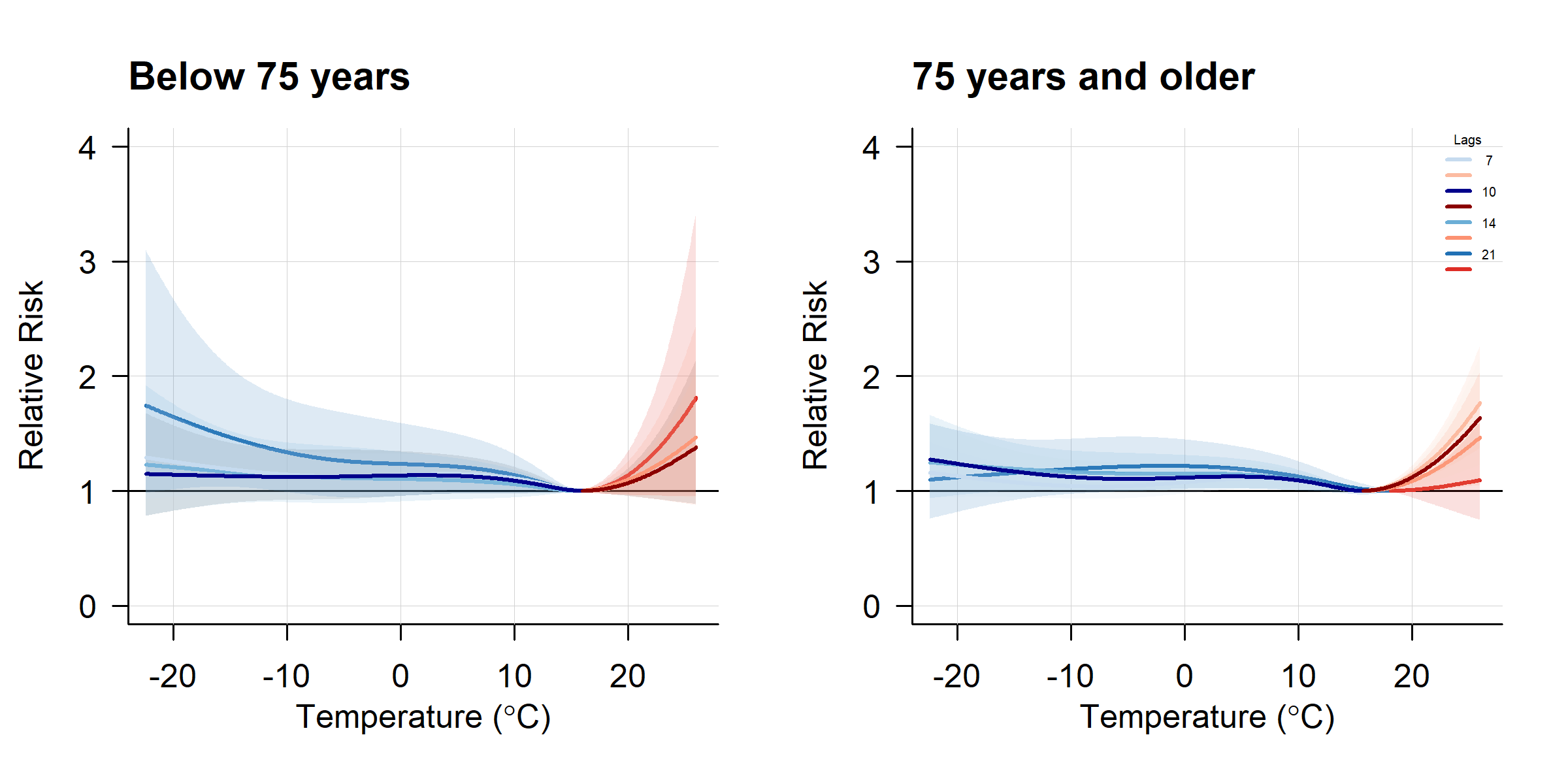


**Figure S2.** Mortality projections by SSP scenario, fixed population, and age group.

Total projected deaths per age group per decade from 2010–2019 to 2090–2099 under Medium Road and Strong Ageing scenarios, including a fixed population scenario.

**
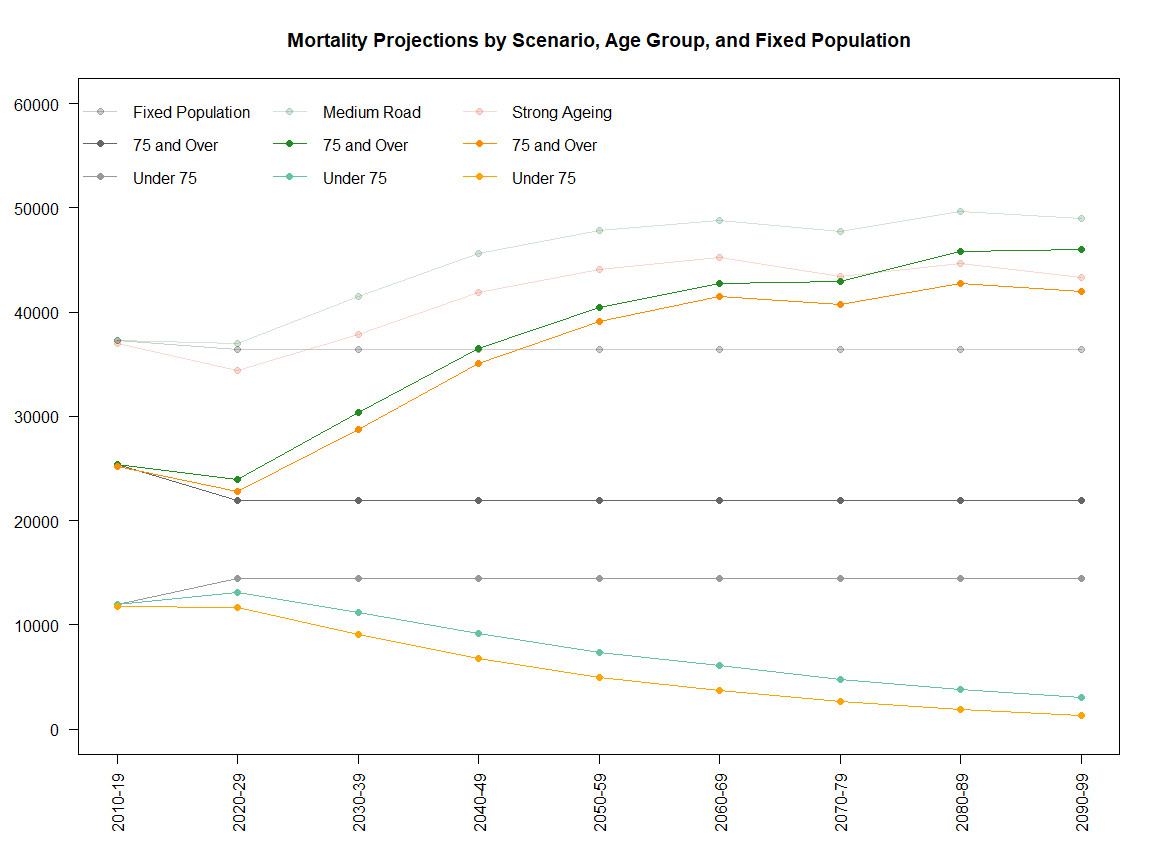
**

**Table S1.** Sensitivity analysis of the cumulative exposure-response associations by age group and lag duration in Oslo.

Area under the curve (AUC) values for cold, heat, and total temperature-attributable risk are presented for each lag structure (7, 10, 14, 21, and 28 days) and age group (<75 and 75+ years). Lag 10 was selected for the main analysis as it represents the longest lag duration at which elevated risks persisted in the older age group while maintaining adequate statistical precision.

| Age group | Lag (days) | Cold AUC | Heat AUC | Total AUC | Standardised AUC   (AUC / Lag) |
| --- | --- | --- | --- | --- | --- |
| <75 | 7 | 43.43 | 10.33 | 53.76 | 7.68 |
| 75+ | 7 | 40.73 | 11.71 | 52.44 | 7.49 |
| **<75** | **10** | **42.29** | **10.34** | **52.63** | **5.26** |
| **75+** | **10** | **42.68** | **11.25** | **53.93** | **5.39** |
| <75 | 14 | 41.22 | 11.73 | 52.95 | 3.78 |
| 75+ | 14 | 42.50 | 11.65 | 54.16 | 3.87 |
| <75 | 21 | 48.17 | 12.78 | 60.96 | 2.90 |
| 75+ | 21 | 43.04 | 10.28 | 53.32 | 2.54 |
| <75 | 28 | 49.97 | 10.66 | 60.63 | 2.17 |
| 75+ | 28 | 56.20 | 9.84 | 66.04 | 2.36 |

**Lag Structure Sensitivity Analysis**

Table S1 presents the results of the sensitivity analysis assessing the impact of lag structure on the area under the curve (AUC) for cold- and heat-attributable mortality, stratified by age group. The total AUC generally increases with longer lag durations; however, when adjusted for lag length via a standardised AUC (i.e., total AUC divided by lag days), a marked decrease is observed with increasing lag.

For instance, among individuals aged 75 and above, the standardised AUC declines from 5.39 at lag 10 to 2.54 at lag 21, and further to 2.36 at lag 28. This trend suggests diminishing marginal contributions of longer lag periods to the overall temperature-attributable mortality signal.

Additionally, the lag of 10 days emerges as the longest duration for which the risk remains higher in the older population compared to those under 75, thereby supporting its selection as a statistically efficient and age-sensitive lag choice in the main analysis.

These findings support the use of a lag of 10 days, balancing biological plausibility, statistical power, and model efficiency in the context of Oslo’s mortality data.

**Table S2a.**  Per‑model bias‑correction diagnostics for EURO‑CORDEX realisations (evaluation period 2000–2018). For each model and scenario, the table reports mean bias before and after bias correction (°C), the mean reduction applied (°C), RMSE before/after (°C) and Pearson correlation before/after (unitless).

| Model | Scenario | Mean Bias  Before | Mean Bias  After | Mean  Reduction | RMSE  Before | RMSE  After | Correlation Before | Correlation  After |
| --- | --- | --- | --- | --- | --- | --- | --- | --- |
| AWI‑CM‑1‑1 (AWI) | RCP4.5 | 1.207 | -0.001 | 1.208 | 6.072 | 5.251 | 0.797 | 0.795 |
| EC‑Earth3 (CC) | RCP4.5 | 2.058 | -0.002 | 2.060 | 6.078 | 5.128 | 0.811 | 0.804 |
| EC‑Earth3‑Veg (LR) | RCP4.5 | 1.154 | 0.004 | 1.150 | 5.911 | 5.156 | 0.810 | 0.801 |
| MPI‑ESM1.2 (LR) | RCP4.5 | -1.647 | 0.011 | -1.658 | 6.106 | 5.264 | 0.782 | 0.794 |
| NorESM2‑LM | RCP4.5 | 2.783 | -0.022 | 2.805 | 6.350 | 5.214 | 0.772 | 0.795 |
| NorESM2‑MM | RCP4.5 | 2.911 | -0.019 | 2.930 | 6.505 | 5.217 | 0.773 | 0.794 |
| AWI‑CM‑1‑1 (AWI) | RCP8.5 | 1.021 | -0.002 | 1.023 | 6.030 | 5.142 | 0.805 | 0.802 |
| EC‑Earth3 (CC) | RCP8.5 | 2.025 | 0.000 | 2.025 | 6.077 | 5.113 | 0.813 | 0.805 |
| EC‑Earth3‑Veg (LR) | RCP8.5 | 0.969 | 0.003 | 0.966 | 5.942 | 5.218 | 0.808 | 0.797 |
| MPI‑ESM1.2 (LR) | RCP8.5 | -1.514 | 0.002 | -1.516 | 5.911 | 5.199 | 0.788 | 0.799 |
| NorESM2‑LM | RCP8.5 | 2.828 | -0.022 | 2.849 | 6.381 | 5.248 | 0.771 | 0.792 |
| NorESM2‑MM | RCP8.5 | 3.067 | -0.023 | 3.090 | 6.496 | 5.132 | 0.779 | 0.800 |
|  |  |  |  |  |  |  |  |  |

**Table S2b.**  Summary bias‑correction diagnostics for EURO‑CORDEX realisations (evaluation period 2000–2018). The table reports median across per‑model time‑mean biases (median of each model’s mean bias over 2000–2018) and diagnostics computed on the ensemble median time series (daily median across model realisations; then mean bias, RMSE and Pearson correlation are computed versus observations). Bias and RMSE values are in °C; correlations are unitless.

|  | Scenario | Median Bias Before | Median Bias After | Median Reduction | RMSE  Before | RMSE  After | Correlation Before | Correlation  After |
| --- | --- | --- | --- | --- | --- | --- | --- | --- |
| Median across per‑model mean biases | RCP4.5 | 1.632 | -0.001 | 1.634 | NA | NA | NA | NA |
| Ensemble median time series diagnostics (daily median across model realisations) | RCP4.5 | 1.691 | 0.100 | 1.591 | 4.761 | 4.185 | 0.855 | 0.859 |
| Median across per‑model mean biases | RCP8.5 | 1.523 | -0.001 | 1.524 | NA | NA | NA | NA |
| Ensemble median time series diagnostics (daily median across model realisations) | RCP8.5 | 1.660 | 0.079 | 1.581 | 4.739 | 4.165 | 0.857 | 0.860 |

**Table S3.** Projected average annual attributable mortality deaths (AN) related to heat and cold per decade under RCP4.5 for different population development scenarios (Climate Only and RCP4.5/Medium Road).

|  | RCP4.5 Climate Only | | | RCP4.5/Medium Road | | |
| --- | --- | --- | --- | --- | --- | --- |
| Decades | Total | Cold | Heat | Total | Cold | Heat |
| 2010-19 | 338 (58-593) | 271 (7-517) | 67 (4-137) | 338 (58-593) | 271 (7-517) | 67 (4-137) |
| 2020-29 | 327 (42-584) | 265 (-1-513) | 62 (2-144) | 334 (48-591) | 270 (5-517) | 64 (3-147) |
| 2030-39 | 327 (42-583) | 264 (-1-510) | 64 (1-159) | 375 (66-654) | 299 (13-567) | 76 (5-183) |
| 2040-49 | 331 (51-581) | 257 (1-498) | 74 (3-164) | 418 (95-709) | 319 (24-599) | 99 (13-208) |
| 2050-59 | 337 (53-590) | 252 (0-487) | 85 (3-223) | 448 (112-752) | 327 (31-608) | 121 (15-298) |
| 2060-69 | 337 (55-586) | 252 (1-487) | 85 (5-182) | 459 (126-760) | 334 (37-618) | 125 (22-248) |
| 2070-79 | 337 (55-589) | 247 (2-476) | 90 (3-207) | 450 (125-744) | 320 (37-587) | 131 (16-275) |
| 2080-89 | 346 (58-605) | 246 (0-478) | 100 (5-235) | 482 (139-796) | 331 (42-612) | 151 (24-324) |
| 2090-99 | 348 (58-598) | 246 (3-477) | 102 (3-214) | 479 (145-778) | 327 (45-598) | 153 (17-291) |

**Table S4** Projected average annual attributable mortality deaths (AN) related to heat and cold per decade under RCP8.5 for different population development scenarios (Climate Only and RCP8.5/Strong Ageing).

|  | RCP8.5 Climate Only | | | RCP8.5/Strong Ageing | | |
| --- | --- | --- | --- | --- | --- | --- |
| Decades | Total | Cold | Heat | Total | Cold | Heat |
| 2010-19 | 338 (57-592) | 270 (6-517) | 68 (4-144) | 336 (57-587) | 268 (6-513) | 68 (4-143) |
| 2020-29 | 335 (49-594) | 264 (0-508) | 71 (2-163) | 318 (54-558) | 249 (5-475) | 69 (3-154) |
| 2030-39 | 337 (48-598) | 261 (0-506) | 76 (1-189) | 353 (71-608) | 270 (16-507) | 84 (5-199) |
| 2040-49 | 338 (50-595) | 257 (-1-499) | 81 (3-206) | 393 (94-664) | 293 (27-546) | 101 (13-240) |
| 2050-59 | 350 (57-616) | 250 (1-482) | 100 (4-234) | 432 (119-719) | 299 (33-551) | 133 (19-286) |
| 2060-69 | 375 (75-653) | 242 (3-463) | 133 (8-314) | 479 (150-791) | 296 (39-541) | 183 (31-400) |
| 2070-79 | 364 (71-623) | 232 (2-447) | 132 (8-271) | 448 (147-722) | 273 (40-498) | 175 (32-328) |
| 2080-89 | 392 (79-670) | 225 (4-432) | 167 (10-346) | 500 (170-803) | 272 (44-494) | 228 (33-429) |
| 2090-99 | 412 (94-690) | 216 (3-417) | 196 (15-377) | 514 (186-803) | 254 (43-463) | 259 (53-448) |

**Table S5.** Net differences in projected average annual attributable mortality due to heat and cold per decade under RCP4.5 for different population development scenarios (Climate Only and RCP4.5/Medium Road), relative to the baseline period 2010-2019. Values represent the mean annual deaths per decade.

|  | RCP4.5 Climate Only | | | RCP4.5/Medium Road | | |
| --- | --- | --- | --- | --- | --- | --- |
| Decades | Total | Cold | Heat | Total | Cold | Heat |
| 2010-19 | Baseline | | | | | |
| 2020-29 | -11 (-45-21) | -5 (-20-11) | -5 (-48-32) | -4 (-39-28) | -1 (-15-15) | -3 (-47-35) |
| 2030-39 | -10 (-38-24) | -7 (-31-12) | -3 (-37-36) | 38 (-2-88) | 29 (2-61) | 9 (-27-60) |
| 2040-49 | -7 (-37-25) | -14 (-43-6) | 7 (-28-37) | 81 (26-141) | 49 (14-95) | 32 (-9-78) |
| 2050-59 | -1 (-46-75) | -19 (-52-5) | 18 (-20-100) | 111 (39-228) | 57 (21-106) | 54 (5-175) |
| 2060-69 | 0 (-39-27) | -19 (-52-7) | 18 (-3-52) | 122 (54-183) | 64 (25-114) | 58 (17-118) |
| 2070-79 | 0 (-49-65) | -24 (-55--1) | 23 (-15-92) | 113 (43-205) | 49 (25-86) | 64 (10-156) |
| 2080-89 | 9 (-40-76) | -24 (-59-3) | 33 (-6-99) | 145 (60-250) | 60 (29-107) | 84 (18-187) |
| 2090-99 | 10 (-53-87) | -25 (-64-6) | 35 (-4-114) | 142 (50-241) | 56 (27-102) | 86 (11-191) |

**Table S6.** Net differences in projected average annual attributable mortality due to heat and cold per decade under RCP8.5 for different population development scenarios (Climate Only and RCP8.5/Strong Ageing), relative to the baseline period 2010-2019.

|  | RCP8.5 Climate Only | | | RCP8.5/Strong Ageing | | |
| --- | --- | --- | --- | --- | --- | --- |
| Decades | Total | Cold | Heat | Total | Cold | Heat |
| 2010-19 | Baseline | | | | | |
| 2020-29 | -3 (-29-36) | -6 (-33-17) | 3 (-26-52) | -18 (-52-22) | -19 (-54-1) | 1 (-29-46) |
| 2030-39 | -1 (-33-43) | -9 (-33-10) | 8 (-13-53) | 18 (-21-65) | 2 (-24-19) | 16 (-8-64) |
| 2040-49 | 0 (-35-52) | -13 (-38-4) | 13 (-31-70) | 58 (18-125) | 25 (5-47) | 33 (-18-105) |
| 2050-59 | 12 (-36-87) | -19 (-51-4) | 32 (-16-114) | 97 (36-190) | 31 (7-56) | 65 (6-165) |
| 2060-69 | 37 (-41-156) | -28 (-70-2) | 65 (1-177) | 143 (47-300) | 28 (-2-53) | 115 (24-263) |
| 2070-79 | 26 (-63-110) | -38 (-91-5) | 64 (0-142) | 113 (6-206) | 5 (-37-39) | 108 (25-198) |
| 2080-89 | 54 (-70-174) | -45 (-103-6) | 99 (4-209) | 164 (16-308) | 4 (-41-42) | 160 (26-292) |
| 2090-99 | 74 (-62-195) | -54 (-124-10) | 127 (10-237) | 178 (17-306) | -13 (-78-44) | 192 (46-308) |

**Table S7.** Net differences in projected decadal attributable fractions (total, heat, and cold) under RCP4.5 for different population development scenarios (Climate Only and RCP4.5/Medium Road), relative to the baseline period 2010-2019.

|  | RCP4.5 Climate Only | | | RCP4.5/Medium Road | | |
| --- | --- | --- | --- | --- | --- | --- |
| Decades | Total | Cold | Heat | Total | Cold | Heat |
| 2010-19 | Baseline | | | | | |
| 2020-29 | -0.29 (-1.23-0.58) | -0.14 (-0.56-0.30) | -0.15 (-1.33-0.88) | -0.11 (-1.04-0.77) | -0.03 (-0.42-0.41) | -0.08 (-1.27-0.94) |
| 2030-39 | -0.29 (-1.05-0.65) | -0.19 (-0.86-0.34) | -0.09 (-1.01-1.00) | 0.91 (-0.06-2.11) | 0.69 (0.06-1.48) | 0.22 (-0.66-1.44) |
| 2040-49 | -0.19 (-1.01-0.69) | -0.38 (-1.18-0.15) | 0.18 (-0.77-1.02) | 1.77 (0.57-3.10) | 1.07 (0.3-2.08) | 0.70 (-0.20-1.72) |
| 2050-59 | -0.02 (-1.26-2.07) | -0.52 (-1.43-0.14) | 0.49 (-0.56-2.74) | 2.32 (0.82-4.76) | 1.19 (0.43-2.22) | 1.13 (0.11-3.65) |
| 2060-69 | -0.01 (-1.08-0.75) | -0.51 (-1.43-0.18) | 0.50 (-0.09-1.44) | 2.50 (1.10-3.75) | 1.31 (0.51-2.33) | 1.19 (0.34-2.42) |
| 2070-79 | -0.01 (-1.36-1.79) | -0.65 (-1.52--0.02) | 0.64 (-0.4-2.52) | 2.36 (0.91-4.30) | 1.03 (0.52-1.80) | 1.34 (0.21-3.28) |
| 2080-89 | 0.24 (-1.11-2.08) | -0.67 (-1.61-0.09) | 0.91 (-0.17-2.72) | 2.92 (1.21-5.04) | 1.22 (0.57-2.16) | 1.70 (0.36-3.77) |
| 2090-99 | 0.28 (-1.44-2.39) | -0.68 (-1.75-0.15) | 0.95 (-0.1-3.14) | 2.89 (1.02-4.92) | 1.14 (0.54-2.08) | 1.75 (0.22-3.89) |

**Table S8.** Net differences in projected decadal attributable fractions (total, heat, and cold) under RCP8.5 for different population development scenarios (Climate Only and RCP8.5/Strong Ageing), relative to the baseline period 2010-2019.

|  | RCP8.5 Climate Only | | | RCP8.5/Strong Ageing | | |
| --- | --- | --- | --- | --- | --- | --- |
| Decades | Total | Cold | Heat | Total | Cold | Heat |
| 2010-19 | Baseline | | | | | |
| 2020-29 | -0.07 (-0.79-0.98) | -0.15 (-0.91-0.45) | 0.08 (-0.71-1.44) | -0.51 (-1.5-0.64) | -0.54 (-1.57-0.02) | 0.03 (-0.83-1.34) |
| 2030-39 | -0.03 (-0.91-1.19) | -0.24 (-0.91-0.28) | 0.21 (-0.36-1.45) | 0.46 (-0.54-1.73) | 0.04 (-0.63-0.49) | 0.42 (-0.21-1.68) |
| 2040-49 | -0.01 (-0.96-1.43) | -0.36 (-1.06-0.11) | 0.36 (-0.85-1.91) | 1.38 (0.43-2.98) | 0.59 (0.12-1.13) | 0.79 (-0.42-2.51) |
| 2050-59 | 0.34 (-0.99-2.4) | -0.53 (-1.39-0.11) | 0.87 (-0.44-3.13) | 2.19 (0.82-4.32) | 0.71 (0.17-1.28) | 1.48 (0.13-3.74) |
| 2060-69 | 1.01 (-1.13-4.29) | -0.78 (-1.92-0.07) | 1.79 (0.03-4.88) | 3.17 (1.03-6.63) | 0.63 (-0.05-1.18) | 2.54 (0.54-5.81) |
| 2070-79 | 0.72 (-1.74-3.04) | -1.04 (-2.5-0.12) | 1.76 (0.01-3.9) | 2.59 (0.14-4.74) | 0.11 (-0.84-0.9) | 2.48 (0.58-4.57) |
| 2080-89 | 1.47 (-1.92-4.78) | -1.24 (-2.84-0.15) | 2.71 (0.1-5.73) | 3.68 (0.35-6.9) | 0.09 (-0.91-0.94) | 3.59 (0.58-6.55) |
| 2090-99 | 2.03 (-1.71-5.35) | -1.48 (-3.41-0.28) | 3.5 (0.28-6.52) | 4.11 (0.39-7.07) | -0.31 (-1.8-1.01) | 4.42 (1.06-7.12) |

**Table S9.** Relative change in excess cold- and heat-attributable mortality under RCP4.5 for different population pathways (Medium Road and Strong Ageing). Percent differences were calculated as: *((RCP4.5/Strong Ageing – RCP4.5/Medium Road) / RCP4.5/Medium Road) × 100*, relative to the 2010–2019 baseline. Values represent total decadal attributable deaths, not average annual mortality per decade.

| Decades |  | RCP4.5/Medium Road | RCP4.5/Strong Ageing | % Difference |
| --- | --- | --- | --- | --- |
| 2010-19 | Cold | 0 | 0 | - |
| 2020-29 |  | -9 | -184 | 1944.4% |
| 2030-39 |  | 286 | 35 | -87.8% |
| 2040-49 |  | 487 | 240 | -50.7% |
| 2050-59 |  | 566 | 324 | -42.8% |
| 2060-69 |  | 637 | 407 | -36.1% |
| 2070-79 |  | 490 | 216 | -55.9% |
| 2080-89 |  | 605 | 286 | -52.7% |
| 2090-99 |  | 560 | 193 | -65.5% |
| 2010-19 | Heat | 0 | 0 | - |
| 2020-29 |  | -31 | -70 | 125.8% |
| 2030-39 |  | 92 | 36 | -60.9% |
| 2040-49 |  | 321 | 255 | -20.6% |
| 2050-59 |  | 542 | 466 | -14.0% |
| 2060-69 |  | 581 | 509 | -12.4% |
| 2070-79 |  | 637 | 538 | -15.5% |
| 2080-89 |  | 843 | 709 | -15.9% |
| 2090-99 |  | 858 | 696 | -18.9% |

**Table S10.** Relative change in excess cold- and heat- related mortality impacts for RCP8.5 using differences combinations of SSPs (Medium Road and strong Ageing). The difference percent was calculated as *((RCP8.5/Strong Ageing – RCP8.5/Medium Road)/RCP8.5/Medium Road)*100* in reference to baseline 2010-2019. Values represent total decadal attributable deaths, not average annual mortality per decade.

| Decades |  | RCP8.5/Medium Road | RCP8.5/Strong Ageing | % Difference |
| --- | --- | --- | --- | --- |
| 2010-19 | Cold | 0 | 0 | - |
| 2020-29 |  | -11 | -185 | 1581.8% |
| 2030-39 |  | 264 | 17 | -93.6% |
| 2040-49 |  | 493 | 246 | -50.1% |
| 2050-59 |  | 555 | 314 | -43.4% |
| 2060-69 |  | 504 | 284 | -43.7% |
| 2070-79 |  | 306 | 50 | -83.7% |
| 2080-89 |  | 331 | 42 | -87.3% |
| 2090-99 |  | 185 | -135 | -173.0% |
| 2010-19 | Heat | 0 | 0 | - |
| 2020-29 |  | 54 | 9 | -83.3% |
| 2030-39 |  | 227 | 159 | -30.0% |
| 2040-49 |  | 404 | 332 | -17.8% |
| 2050-59 |  | 743 | 653 | -12.1% |
| 2060-69 |  | 1266 | 1150 | -9.2% |
| 2070-79 |  | 1224 | 1077 | -12.0% |
| 2080-89 |  | 1828 | 1602 | -12.4% |
| 2090-99 |  | 2228 | 1915 | -14.0% |
